# Microbes Drive Straw Decomposition and Microbial Metabolism in Mollisols with Different Straw Return Rates

**DOI:** 10.3390/microorganisms14091929

**Published:** 2026-09-01

**Authors:** Guiying Cui, Peng Zhang, Qian Chen, Jiuming Zhang, Qingyi Wang, Shanshan Zhang, Yiming Shi, Zhidan Zhang, Yang Wang

**Affiliations:** 1 College of Resource and Environmental Science, Jilin Agricultural University, Changchun 130118, China; 18354028568@163.com (G.C.); zhangpeng@jlau.edu.cn (P.Z.); 15763104102@163.com (Q.C.); s2878457308@163.com (Y.S.); 2 Heilongjiang Academy of Agricultural Sciences, Institute of Soil Fertilizer and Environment Resources, Harbin 150086, China; zjm_8049@163.com (J.Z.); 18724316315@163.com (Q.W.); 3 Science and Technology Park, China Grain Reserves Northern Agricultural Development Co., Ltd., Nenjiang 161499, China; 4 School of Environmental and Natural Sciences, Bangor University, Bangor LL57 2UW, UK; shz25prb@bangor.ac.uk

**Keywords:** extracellular enzymes, microbial succession, straw decomposition, straw return rate, untargeted metabolomics

## Abstract

The process of straw decomposition is highly complex and is regulated by a multitude of interacting factors. However, how the straw return rate influences the metabolic byproducts, extracellular enzymes, and microbial communities during straw decomposition remains an unresolved question. To address this, we integrated straw and soil chemistry, amplicon sequencing, untargeted metabolomics, and enzyme assays across four straw return rates (no return, 1/3, 1/2, and the Full treatment) at 30 and 90 d. Straw mass loss was greatest under the 1/2 return treatment at 90 d, reaching 53.7%, whereas soil organic carbon (SOC) and total nitrogen (N) were highest under the 1/3 return, exceeding the control by 17.6% and 17.2%, respectively. This indicates that decomposition and short-term soil C and N accumulation were decoupled. Microbial communities underwent clear temporal turnover, and hydrolytic enzymes increased from 30 to 90 d and were positively associated with mass loss. In contrast, oxidative enzymes showed no positive association with decomposition. Metabolomic profiles shifted from early-stage labile compounds to later-stage aromatic and phenolic compounds, and straw and soil metabolomes were closely coupled in a stage-specific manner (Procrustes M^2^ = 0.15, *p* = 0.001; Mantel r = 0.69, *p* = 0.001). Mantel tests further indicated that the metabolome was significantly associated with return rate and SOC. Partial least squares path modeling revealed that the direct and indirect pathways linking return rate, metabolome, enzymes, and decomposition were reorganized between 30 and 90 d. Overall, straw return rate influenced decomposition through stage-dependent biochemical and microbial changes, and straw mass loss and short-term soil C and N accumulation were decoupled, responding nonlinearly and peaking under different return rates.

## 1. Introduction

In the Mollisol region of Northeast China, straw incorporation serves as a foundational practice for maintaining soil organic carbon (SOC) and nutrient cycling, and global syntheses confirm that straw return can substantially rebuild SOC stocks in croplands [1]. Returning crop residues replenish carbon (C) and nitrogen (N), stimulates microbial activity, and can slow the long-term decline of soil fertility caused by intensive cultivation [2,3]. Straw decomposition is a microbially driven process governed by the interplay between substrate chemistry, microbial community succession, and extracellular enzyme production [4]. It typically proceeds in two stages: an early phase in which bacteria and hydrolytic enzymes mineralize labile compounds such as sugars and amino acids, followed by a later phase in which fungi and oxidative enzymes degrade recalcitrant aromatic and phenolic residues [5,6]. This bacteria-to-fungi and hydrolase-to-oxidase succession has been documented in maize residues in Northeast China [7] and in forest leaf litter [8], and it underpins the litter-to-soil continuum through which residue C is either mineralized as CO_2_ or stabilized into SOC [9]. Accumulating evidence further indicates that fungi and microbial necromass are more important than bacteria in retaining residue-derived C [10,11]. Nevertheless, most of these studies characterized decomposition under a single, fixed residue input level [12]; how the timing, intensity, and consequences of this microbial and enzymatic succession shift across contrasting straw return rates therefore remains unknown.

Recent advances in untargeted metabolomics provide a direct window into the substrate environment that microorganisms and enzymes respond to [13]. Metabolomic studies have shown that labile metabolites (e.g., sugars, amino acids) dominate the early decomposition phase, whereas the accumulation of aromatic and phenolic metabolites signals the transition to recalcitrant residue degradation [14,15,16]. For instance, untargeted metabolomics has been used to link shifts in soil metabolites to microbial community changes under different tillage practices, demonstrating its power to resolve substrate–microbe relationships [17,18]. Integrating metabolomics with microbial community and enzyme data would create a microbe-metabolite-enzyme coupling framework capable of mechanistically explaining why different return rates lead to divergent decomposition and C retention outcomes [17]. However, such multi-omics coupling during straw decomposition, and especially its dependence on return rate, has rarely been examined in Mollisols [19].

A key knowledge gap remains: does increasing the straw return rate accelerate early decomposition at the expense of later SOC accumulation? That is, does a trade-off exist between rapid mineralization and C retention? And is this trade-off linear, or does it exhibit a dose threshold? Prior work hints at nonlinearity: repeated litter inputs promote stable SOC formation by increasing fungal dominance and carbon use efficiency [11], whereas full straw return can produce contrasting outcomes for necromass-derived C [20], and stoichiometric imbalances between microorganisms and their resources can further constrain decomposition [21]. These results suggest that microbial communities may respond to residue input rates in a threshold-like manner, with low and moderate rates enriching saprotrophic fungi and fungal necromass C, whereas the Full treatment may suppress these benefits [20]. Resolving this requires simultaneously tracking substrate metabolites, microbial communities, and enzymes across decomposition stages under contrasting return rates.

In this study, we conducted a 90-day decomposition experiment using four straw return rates (no return, 1/3, 1/2, and the Full treatment) in a Mollisol. We integrated straw and soil chemistry, amplicon sequencing, untargeted metabolomics, and extracellular enzyme assays at 30 and 90 days. We define the decomposition-retention trade-off as a condition in which treatments that maximize residue mass loss do not simultaneously maximize short-term SOC retention. Our objective was to elucidate how straw return rate reshapes microbe-metabolite-enzyme coupling and governs the balance between decomposition and soil C retention. We hypothesized that (1) straw return rate regulates decomposition nonlinearly, with an intermediate rate maximizing residue mass loss while a lower rate favors short-term SOC retention, indicating a decoupling rather than a simple trade-off between decomposition and C accumulation; (2) decomposition is accompanied by a stage-specific shift from labile, bacteria-associated metabolism at 30 d to aromatic/phenolic, fungi-associated metabolism at 90 d, reflected in both enzyme activities and metabolite profiles; and (3) metabolome-microbiome coupling is the key mediating mechanism linking return rate to divergent C and N dynamics, with residue mass loss not scaling proportionally to short-term soil C retention.

## 2. Materials and Methods

### 2.1. Study Site and Experimental Design

This study, supported by the Heilongjiang Academy of Agricultural Sciences, was conducted based on a long-term field experiment established in 2012 at the Science Park Experimental Station of Sinograin North Company in Nenjiang, Heilongjiang Province, Northeast China (49°33′35″ N, 125°27′05″ E). Based on the USDA soil taxonomy, the soil at the experimental site is categorized as a typical Mollisol (black soil). The study area features a temperate continental monsoon climate, with a mean annual temperature of 0.8–1.4 °C, an annual precipitation of 450 mm, a frost-free period of approximately 115 days, and an effective accumulated temperature of 2150 °C d. The initial topsoil (0–20 cm) basic physicochemical properties were as follows: soil organic matter 45.9 g kg^−1^, total TN 2.5 g kg^−1^, total phosphorus (TP) 2.0 g kg^−1^, total potassium (TK) 22.7 g kg^−1^, alkali-hydrolyzable N 211.9 mg kg^−1^, available P 78.6 mg kg^−1^, and available K 226.7 mg kg^−1^.

The field has been continuously planted with maize (Zea mays cv. Demeiya No.2) since 2012. After annual autumn harvesting, maize straw was mechanically crushed and incorporated into the soil via rotary tillage. Each plot covered an area of 39 m^2^ with four replicates per treatment. Uniform fertilization was applied across all treatments, consisting of 150 kg hm^−2^ N, 75 kg hm^−2^ P_2_O_5_, and 75 kg hm^−2^ K_2_O. The four straw return treatments were established from 2018 to 2020: (1) CK: only root stubble return without straw incorporation; (2) 1/3: one-third straw return (3000 kg hm^−2^ dry straw); (3) 1/2: half straw return (4500 kg hm^−2^ dry straw); (4) 1: full straw return (9000 kg hm^−2^ dry straw). All fertilizers were applied as basal fertilization before sowing.

### 2.2. In Situ Litterbag Decomposition Experiment

An in situ litterbag method was used to characterize the dynamic decomposition of maize straw. Maize straw was collected after autumn harvesting, air-dried, cut into 5–7 cm segments, and oven-dried at 60 °C for 24 h to constant weight to eliminate moisture interference. The initial straw C, N, and lignocellulose components were determined before incubation. Briefly, 15.00 g of oven-dried straw was sealed in a small nylon litterbag (10 cm × 15 cm, 100-mesh, aperture ≈ 0.15 mm). This mesh size excludes soil macrofauna while allowing free penetration of soil microorganisms, water, and fine soil particles. Each small straw bag was placed inside an outer large nylon bag (15 cm × 20 cm, 20-mesh, aperture ≈ 0.8 mm) filled with 200 g of sieved topsoil collected from the corresponding field plot. The soil inside the outer bag was compacted to approximate the field bulk density to simulate in-situ soil microenvironments. Seven nested litterbags were connected in series with nylon ropes and buried in the furrow layer (0–20 cm) of each plot with marked tags at both ends. During conventional field tillage, all litterbags were temporarily excavated and re-buried immediately after tillage to ensure that straw materials experienced natural seasonal freeze–thaw cycles and consistent field environmental conditions throughout the decomposition period.

### 2.3. Sample Collection and Processing

Soil and straw samples were obtained on days 30 and 90 after straw incorporation, representing the early and middle decomposition stages. Each treatment contained 28 litterbags (4 replicate strings × 7 bags per string). At each sampling time, four replicate string sets were randomly retrieved per plot, immediately stored in dry-ice insulated containers, transported to the laboratory. For each treatment, two replicate straw sets were flushed with tap water to remove surface-adhered soil particles, further rinsed with deionized water three times, oven-dried at 60 °C to a constant weight, and ground for determining straw residual mass, decomposition loss, and straw C and N release characteristics. The other two replicate straw samples were stored at −80 °C in a freezer for subsequent analysis of straw surface enzyme activity, microbial community abundance, and metabolomic profiling. All soil samples were sieved through a 2 mm mesh and partitioned for enzyme activity determination, metabolome analysis, and high-throughput sequencing.

### 2.4. Measurements and Analytical Methods

#### 2.4.1. Soil and Straw Carbon and Nitrogen Contents

Total C and N in soil and straw were quantified via dry combustion using an elemental analyzer. Before measurement, samples were pulverized to pass a 0.15 mm sieve and loaded into tin capsules. Since the Mollisol samples contained no inorganic C, the determined total C was interpreted as SOC.

#### 2.4.2. Straw Decomposition Characteristics

Straw decomposition dynamics were determined using the mass loss method. The straw residual dry weight at each sampling time was recorded to calculate the straw mass remaining, mass loss rate, and C and N release rates. A first-order kinetic model was applied to determine the straw decomposition parameters:

(1) $$W_{t}/W_{0}=e^{-\mathrm{kt}}$$

where *W*_0_ and *W*_t_ represent the initial straw dry weight and residual dry weight at time t, respectively; k is the decomposition rate constant; and t is decomposition days. The straw decomposition half-life ($t_{1/2}$) was calculated as $t_{1/2}\;=\;\ln\;2/k$.

#### 2.4.3. Microbial Community Composition

Genomic DNA was isolated from both the straw surface and soil subsamples (0.2–0.5 g) utilizing the MagBeads FastDNA Kit for Soil (Cat. 116564384, MP Biomedicals, Santa Ana, CA, USA) according to the manufacturer’s protocol. The NanoDrop NC2000 spectrophotometer (Thermo Fisher Scientific, Waltham, MA, USA) and 0.8% agarose gel electrophoresis were subsequently used to evaluate DNA quality and concentration. Shanghai Personal Biotechnology Co., Ltd. (Shanghai, China) conducted the high-throughput amplicon sequencing on the Illumina NovaSeq platform (using the NovaSeq 6000 SP Reagent Kit, 500 cycles; Illumina, San Diego, CA, USA). The bacterial 16S rRNA gene V3-V4 region was amplified using primers 338F (5′-ACTCCTACGGGAGGCAGCA-3′) and 806R (5′-GGACTACHVGGGTWTCTAAT-3′). The fungal ITS1 region was amplified using primers ITS5 (5′-GGAAGTAAAAGTCGTAACAAGG-3′) and ITS2 (5′-GCTGCGTTCTTCATCGATGC-3′). We performed PCR amplification in a 25 µL reaction mixture containing NEB Q5 High-Fidelity DNA Polymerase (NEB, M0491L). The thermal cycling conditions for bacterial samples included an initial denaturation at 98 °C for 5 min, followed by 25 cycles of denaturation at 98 °C for 30 s, annealing at 52 °C for 30 s, and extension at 72 °C for 45 s, with a final extension step at 72 °C for 5 min. For fungi, annealing was performed at 55 °C for 45 s over 30 cycles. Purification and quantification of the PCR amplicons were carried out using the Quant-iT PicoGreen dsDNA Assay Kit (Invitrogen, Carlsbad, CA, USA, P7589). They were then pooled in equal amounts and subjected to paired-end sequencing (2 × 250 bp). The TruSeq Nano DNA LT Library Prep Kit (Illumina, San Diego, CA, USA) was employed for library construction. The raw reads were processed through a bioinformatic pipeline consisting of demultiplexing, quality filtering, denoising, merging, and chimera filtering via the DADA2 (version 2024.5) algorithm within QIIME2 (version 2024.5) [22,23], ultimately yielding amplicon sequence variants (ASVs). For taxonomic assignment, the generated ASVs were classified against the SILVA 138 database (for bacteria) and the UNITE 9.0 database (for fungi) [24,25] using the classify-sklearn naïve Bayes classifier. Bacterial and fungal samples were subsampled to depths of 20,159 and 45,444 sequences, respectively. Alpha diversity metrics (Shannon and Simpson) were computed using QIIME2(version 2024.5). Bray–Curtis and Jaccard distance matrices were employed to evaluate beta diversity and visualized through principal coordinate analysis (PCoA). The PERMANOVA and PERMDISP tests were conducted to assess compositional differences among the groups.

#### 2.4.4. Straw Enzyme Activities

Five extracellular enzymes involved in C and N cycling were quantified in maize straw residues: C-degrading β-glucosidase (BG), N-degrading N-acetyl-β-D-glucosaminidase (NAG) and leucine aminopeptidase (LAP), and polyphenol oxidase (PPO, abbreviated PO in the calculations) and peroxidase (POD) involved in phenolic oxidation. Crude enzyme solutions were extracted by homogenizing 0.1 g of sample with 1 mL of extraction buffer on ice, followed by centrifugation at 15,000× *g* (BG, NAG), 8000× *g* (POD, LAP), or 12,000 rpm (PPO) for 10 min at 4 °C, according to the respective kit instructions. All enzyme activities were then determined via commercial spectrophotometric microplate kits (Shanghai Jingkang Biological Engineering Co., Ltd., Cat NAG-W48S-N(1620), BGC-W48-N(1620), PO-W96S-N(1620), POD-W96-N(1621), LAP-W96S-N(1620), Shanghai, China) in 96-well microplates, following the manufacturer’s standard microscale colorimetric protocols. Two assay types were adopted: (1) Colorimetric quantification with standard calibration curves (BG, NAG): Activities were calculated based on the linear regression equation of p-nitrophenol. One unit (U) of BG/NAG activity was defined as the production of 1 nmol p-nitrophenol per gram sample per minute. For LAP, activity was calculated based on the molar extinction coefficient of p-nitroaniline. One LAP unit represented 1 nmol p-nitroaniline generated per gram sample per minute. (2) Direct absorbance change quantification (PPO, POD): No standard curve was required. For PPO, one activity unit represented a 0.001 absorbance increase at 490 nm per gram sample per minute; for POD, one unit corresponded to a 0.005 absorbance shift at 470 nm per mL reaction system per gram sample per minute. Brief calculation formulas and fixed incubation parameters are shown in Supplementary Table S1. In brief, incubation lasted 30 min for BG, NAG and PPO, 2 min for LAP, and 1 min for POD at 37 °C. The corresponding reaction system volumes (V2), sample loading volumes (V1), and dilution factors (DF) are listed in Table S1.

#### 2.4.5. Untargeted Metabolomic Analysis

UHPLC-MS/MS (ultra-high-performance liquid chromatography coupled with tandem mass spectrometry) was utilized for the untargeted metabolomic profiling of both straw residue and bulk soil. For straw samples, 40 mg of pulverized material was homogenized in 300 µL of chilled methanol-acetonitrile-water (2:2:1, *v*/*v*/*v*) containing 5 ppm 2-chlorophenylalanine as an internal standard. Meanwhile, 100 mg of each soil sample (straw-associated soil and bulk soil) was processed with 300 µL of pre-cooled methanol spiked with 5 ppm 2-chlorophenylalanine. After vortexing for 30 s, all samples were subjected to two rounds of grinding in a high-throughput tissue grinder (55 Hz, 60 s). They were then ultrasonicated for 10 min, kept at −20 °C for 30 min, and subsequently centrifuged at 12,000 rpm for 10 min under 4 °C. The resultant supernatant was passed through a 0.22 μm membrane prior to instrumental analysis. Finally, equal volumes (10–20 µL) of each filtrate were pooled together to create the quality control (QC) samples.

UHPLC-MS analyses were carried out on a Vanquish Flex UHPLC system (Thermo Fisher Scientific) outfitted with an ACQUITY UPLC HSS T3 column (100 A, 1.8 µm, 2.1 mm × 100 mm). Separation was performed at 40 °C, with an autosampler temperature of 8 °C, a flow rate of 0.4 mL/min, and a 2 µL injection volume. The mobile phases consisted of solvent A (0.1% formic acid in water) and solvent B (acetonitrile containing 0.1% formic acid). The gradient elution program was set as follows: 0–1 min, 5% B; 1–4.7 min, 5–95% B; 4.7–6 min, 95% B; 6–6.1 min, 95–5% B; 6.1–8.5 min, 5% B.

We performed mass spectrometric analysis on an Orbitrap Exploris 120 mass spectrometer (Thermo Fisher Scientific) equipped with a HESI source that operated in both positive and negative ion modes. The DDA parameters were configured as follows: a spray voltage of 3.5 kV (positive) and −3.0 kV (negative); sheath and auxiliary gases at 40 and 10 arb, respectively; capillary and auxiliary gas heater temperatures at 320 °C and 300 °C, respectively; a full MS resolution of 60,000; an m/z scan range of 70–1000; an AGC target set to “Standard”; and a maximum injection time of 100 ms. For MS/MS fragmentation, the top four precursor ions were isolated at a resolution of 15,000 using a 30% normalized HCD collision energy, and the dynamic exclusion interval was maintained at 4 s. To ensure instrument stability, QC samples were interspersed every 6–12 injections.

We processed the raw mass spectrometry data (.raw) using MS-DIAL software (v4.9.221218) to perform peak extraction, alignment, filtration, and metabolite annotation [26,27]. Metabolite features with a missing rate exceeding 50% within any group were excluded, and the remaining missing values were imputed. Metabolite identification was accomplished by searching against the Personalbio Next-Generation Metabolomics Database (PSNGM), along with standard in-house libraries and external databases such as mzCloud, LIPID MAPS, HMDB, MoNA, NIST_2020_MSMS, and AI-predicted MS/MS spectral libraries (setting the MS1 tolerance to 0.01 Da, MS2 tolerance to 0.05 Da, and identification score cutoff to 70). Multivariate statistical models, including PCA and OPLS-DA, were constructed in R (v4.6.1) through the ropls package (version 1.44.0) [28]. Differentially expressed metabolites (DEMs) were screened based on an OPLS-DA VIP > 1.0, a |log2 fold change| > 1, and a Benjamini–Hochberg-corrected *p*-value < 0.05 (Student’s *t*-test). The reliability of the OPLS-DA model was validated using 200 random permutation tests. Finally, KEGG pathway enrichment analysis was carried out to pinpoint metabolic pathways closely associated with the straw decomposition process.

### 2.5. Statistical Analysis

Microsoft Excel 2016 and R software (v4.6.1) were employed for data arrangement and visualization. Prior to statistical analysis, the normality (Shapiro–Wilk test) and homoscedasticity (Levene’s test) of all datasets were assessed. Treatment differences at each sampling time were evaluated via one-way ANOVA combined with Tukey’s HSD post-hoc test; in cases where the homogeneity of variance assumption was violated, Welch’s ANOVA was applied (*p* < 0.05). For variables that failed to satisfy the normality assumption even after log-transformation (e.g., the relative abundance of microbial taxa), the Kruskal–Wallis test was utilized, followed by Dunn’s test with Benjamini–Hochberg adjustment. Differences between the two decomposition stages (30 d vs. 90 d) were examined using independent Student’s *t*-tests. A two-way ANOVA was carried out to assess the interactive effects of straw return rate and decomposition duration on soil properties, enzyme activities, and microbial metrics.

Using the QIIME2 platform, microbial α-diversity was determined from the ASV matrices. For β-diversity, Bray–Curtis distance-based principal coordinate analysis (PCoA) was employed for visualization, while differences in community structures across treatments were evaluated by PERMANOVA (with 999 permutations) utilizing the vegan package(v2.7.5) in R [29]. PERMDISP was used to verify whether community differences were attributable to dispersion effects. Differential abundance analysis was performed using ANCOM-BC2 in QIIME2 [30]. Procrustes analysis and Mantel test were used to evaluate the coupling between straw and soil metabolomes. Spearman correlation analysis was applied to establish microbe-metabolite-enzyme association networks, with thresholds set at |r| > 0.6 and Benjamini–Hochberg-adjusted *p* < 0.05.

We conducted partial least squares path modeling (PLS-PM) via the plspm package(v0.4.9) in R to determine the direct and indirect factors driving straw decomposition at both 30 d and 90 d sampling points [31]. Five latent variables were incorporated into this model: straw return rate, metabolome (captured by the first two principal components from PCA of metabolite profiles), hydrolytic enzymes (averaged z-score-standardized values for BG, NAG, and LAP), oxidative enzymes (averaged z-score-standardized values for PPO and POD), and decomposition rate. All variables were converted to z-scores, and path coefficients together with 95% confidence intervals were computed based on 1000 bootstrap resampling iterations. The R-squared (R^2^) value was obtained for each endogenous latent variable, and the model’s goodness-of-fit (GOF) was assessed, with a GOF exceeding 0.36 interpreted as large [32].

## 3. Results

### 3.1. Soil Carbon and Nitrogen Content

Straw return rates and incorporation time significantly influenced SOC, TN, and their stoichiometric ratios (Figure S1). At 30 days, SOC contents showed limited differences among treatments. However, by 90 days, the 1/3 straw return treatment exhibited significantly higher SOC content than the CK, 1/2, and Full treatments (Figure S1a). This trend was further corroborated by the SOC increment results: the 1/3 treatment displayed the greatest positive SOC increment at 90 days, whereas the 1/2 treatment resulted in a negative SOC increment (Figure S1b). Similarly, TN contents at 90 days were significantly higher than those at 30 days. Specifically, TN was highest under the 1/3 treatment at 90 days, followed by the CK treatment, while the 1/2 and Full treatments were significantly lower (Figure S1c). Interestingly, the soil C/N ratio significantly increased over time (*p* < 0.001), with values at 90 days being markedly higher than at 30 days across all treatments. However, at each specific time point, no significant differences in soil C/N ratios were detected among the four straw return treatments (Figure S1d).

### 3.2. The Decomposition Rate, Carbon and Nitrogen Content in Straw

**Figure 1:**
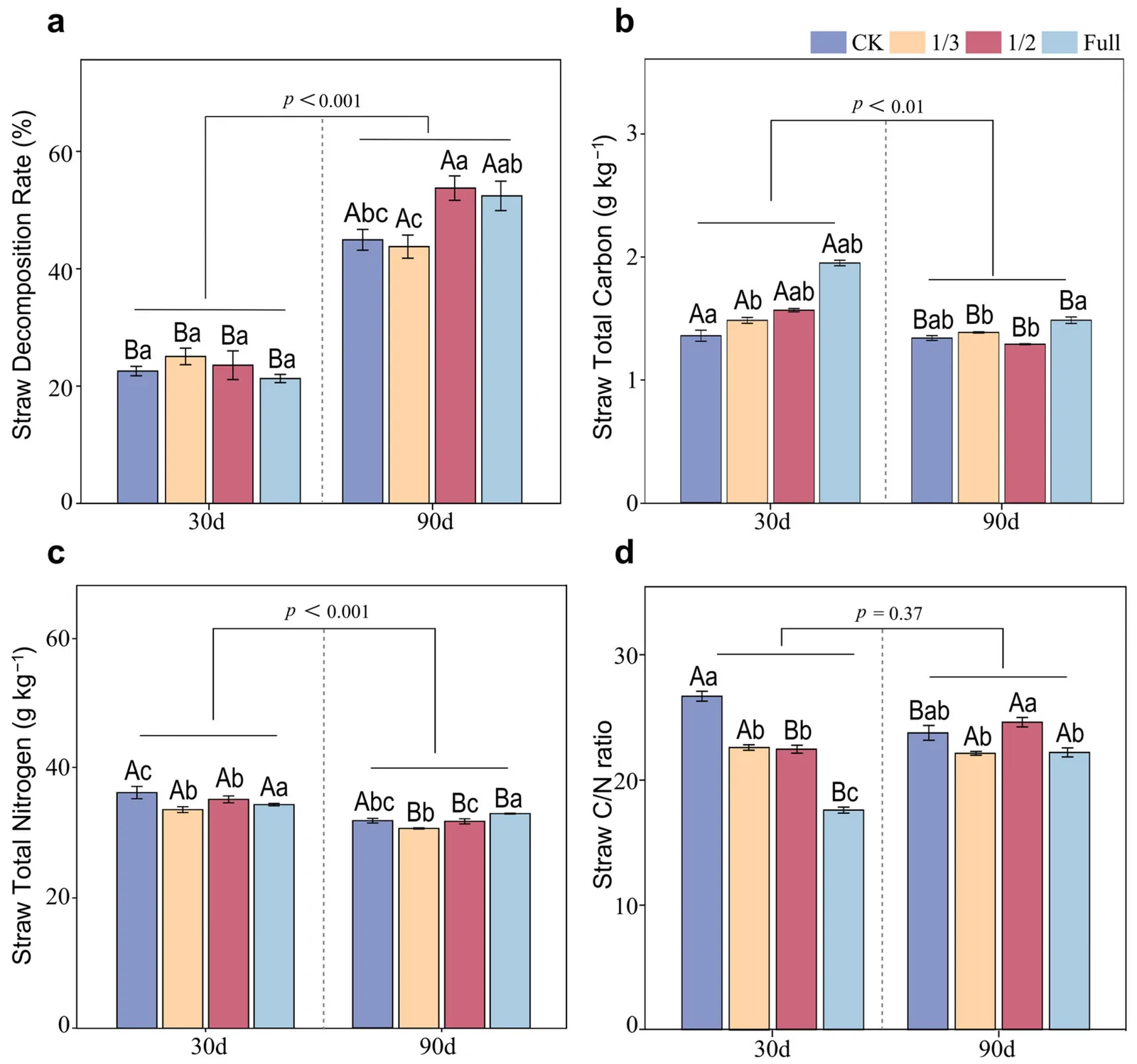
Changes in straw decomposition rate (**a**), straw total carbon (**b**), straw total nitrogen (**c**), and straw C/N ratio (**d**) in Mollisols under different straw return rates at 30 and 90 days post-incorporation. The treatments CK, 1/3, 1/2, and the Full treatment represent the control (no straw return), one-third, half, and full straw return rates, respectively. Bars represent means ± standard error (*n* = 4). Within each sampling time point (30 d or 90 d), different lowercase letters above the columns indicate significant differences among treatments (Tukey’s HSD test, *p* < 0.05); within the same straw incorporation treatment, different uppercase letters denote significant differences between the two sampling times (Student’s *t*-test, *p* < 0.05).

Straw decomposition was strongly influenced by incubation time and, at the later stage, by straw return rate (Figure 1a). At 30 d, mass loss remained relatively low, ranging from approximately 21% to 25%, and did not differ significantly among treatments. By 90 d, decomposition had increased substantially compared with 30 d (*p* < 0.001) and varied among return rates, peaking under the 1/2 return rate, followed by the Full treatment, whereas CK and the 1/3 return rate showed the lowest values. These results indicate that decomposition did not increase monotonically with straw input but instead reached a maximum at an intermediate return rate.

Residue quality changed markedly during decomposition (Figure 1b–d). Straw total C declined significantly over time, and at 30 d it was highest under the Full treatment, consistent with the larger initial C input. Straw total N also decreased with incubation time (*p* < 0.001). In contrast, the straw C/N ratio showed no significant overall temporal effect (*p* = 0.37), but it decreased with increasing return rate at 30 d, from approximately 26 under CK to approximately 17.5 under the Full treatment. Collectively, these patterns suggest that straw return rate influenced decomposition and soil C/N dynamics in a nonlinear and time-dependent manner.

### 3.3. Succession of Bacterial and Fungal Communities

**Figure 2:**
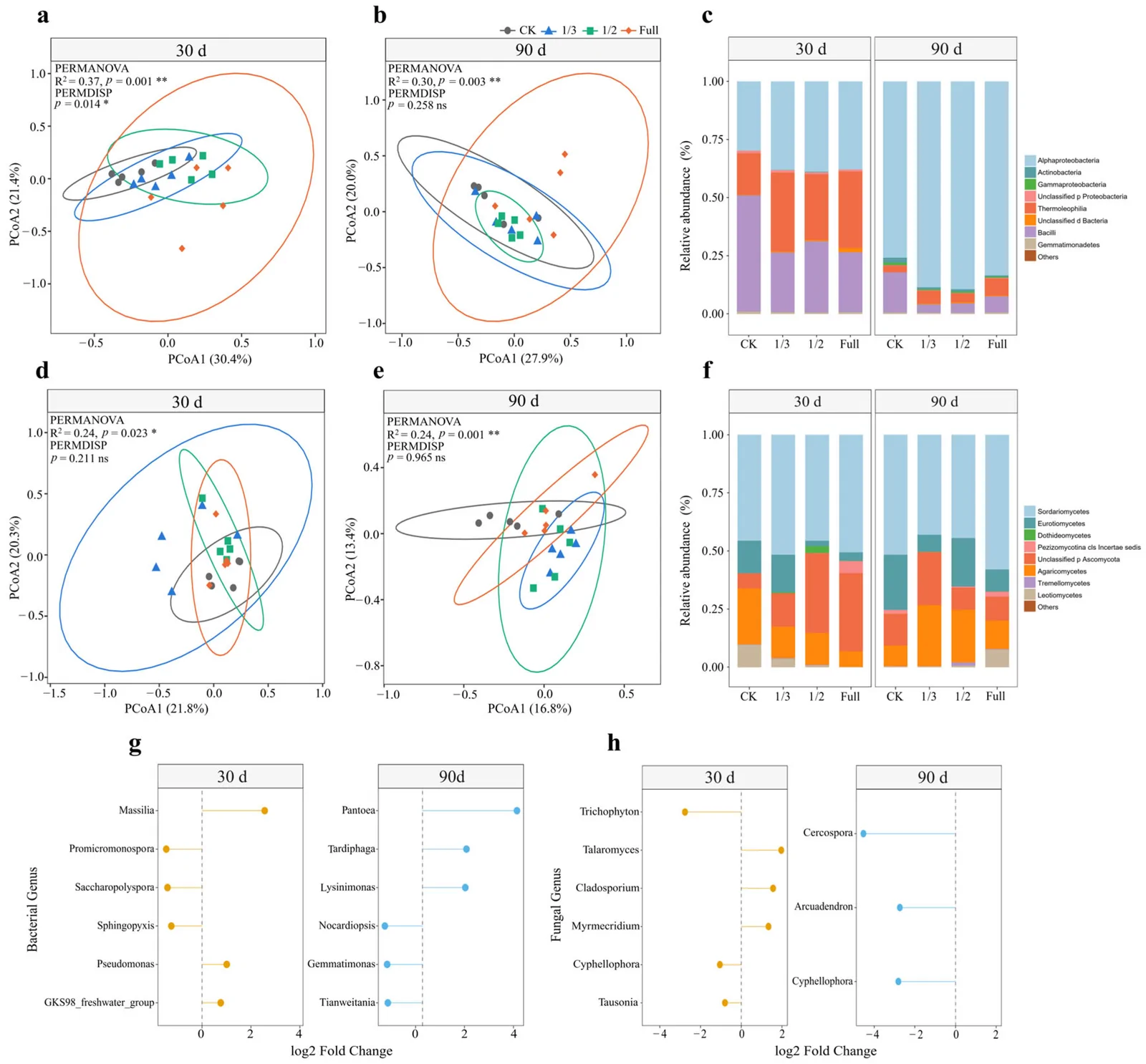
Succession of bacterial and fungal community structures in Mollisols under different straw return rates at 30 and 90 days post-incorporation. (**a**,**b**) Principal coordinate analysis (PCoA) of bacterial beta diversity based on Bray–Curtis distances at 30 and 90 days, respectively. (**d**,**e**) PCoA of fungal beta diversity. The effects of treatments on community structure were evaluated using PERMANOVA, and dispersion was assessed by PERMDISP. Relative abundance of dominant bacterial (**c**) and fungal (**f**) classes at 30 and 90 days. Log2 fold changes in significantly enriched or depleted genera under the Full treatment relative to CK at 30 d (orange dots) and 90 d (blue dots) for bacterial (**g**) and fungal (**h**) communities, respectively (FDR < 0.05). Data are based on four replicates (*n* = 4). Asterisks indicate statistical significance: * *p* < 0.05, ** *p* < 0.01.

Bacterial and fungal community composition differed significantly among treatments and between sampling times (Figure 2a,b,d,e). PERMANOVA showed significant differences in both bacterial and fungal communities at 30 d and 90 d. PERMDISP was non-significant for most comparisons, indicating that the ordination patterns mainly reflected compositional turnover rather than differences in dispersion. However, the bacterial comparison at 30 d showed significant dispersion heterogeneity (*p* = 0.014), suggesting that this early-stage separation should be interpreted with caution. Full PERMANOVA and PERMDISP statistics are presented in Table S4.

At the class level, bacterial communities underwent a pronounced shift from 30 to 90 d, with Alphaproteobacteria increasing in relative abundance at the later stage, whereas Actinobacteriota and other early-stage taxa declined (Figure 2c, Table S2). Fungal communities remained dominated by Sordariomycetes but showed changes in the relative abundance of Eurotiomycetes, Agaricomycetes and unclassified Ascomycota (Figure 2f, Table S3). Differential-abundance analysis identified stage-specific responders under full straw return relative to CK (Figure 2g,h): at 30 d, Massilia was enriched among bacteria, whereas Talaromyces and Cladosporium were enriched among fungi; at 90 d, Pantoea, Tardiphaga and Lysinimonas were enriched among bacteria, while Cercospora was enriched among fungi.

Alpha diversity also responded to straw return and incubation time (Figure S2). Different lowercase letters indicate significant differences among treatments at each sampling time, and different uppercase letters indicate significant differences between the two sampling times within each treatment (Figure S2). Overall, these results show that straw return reshaped both the taxonomic composition and diversity of bacterial and fungal communities in a stage-dependent manner.

### 3.4. Extracellular Enzyme Activities

**Figure 3:**
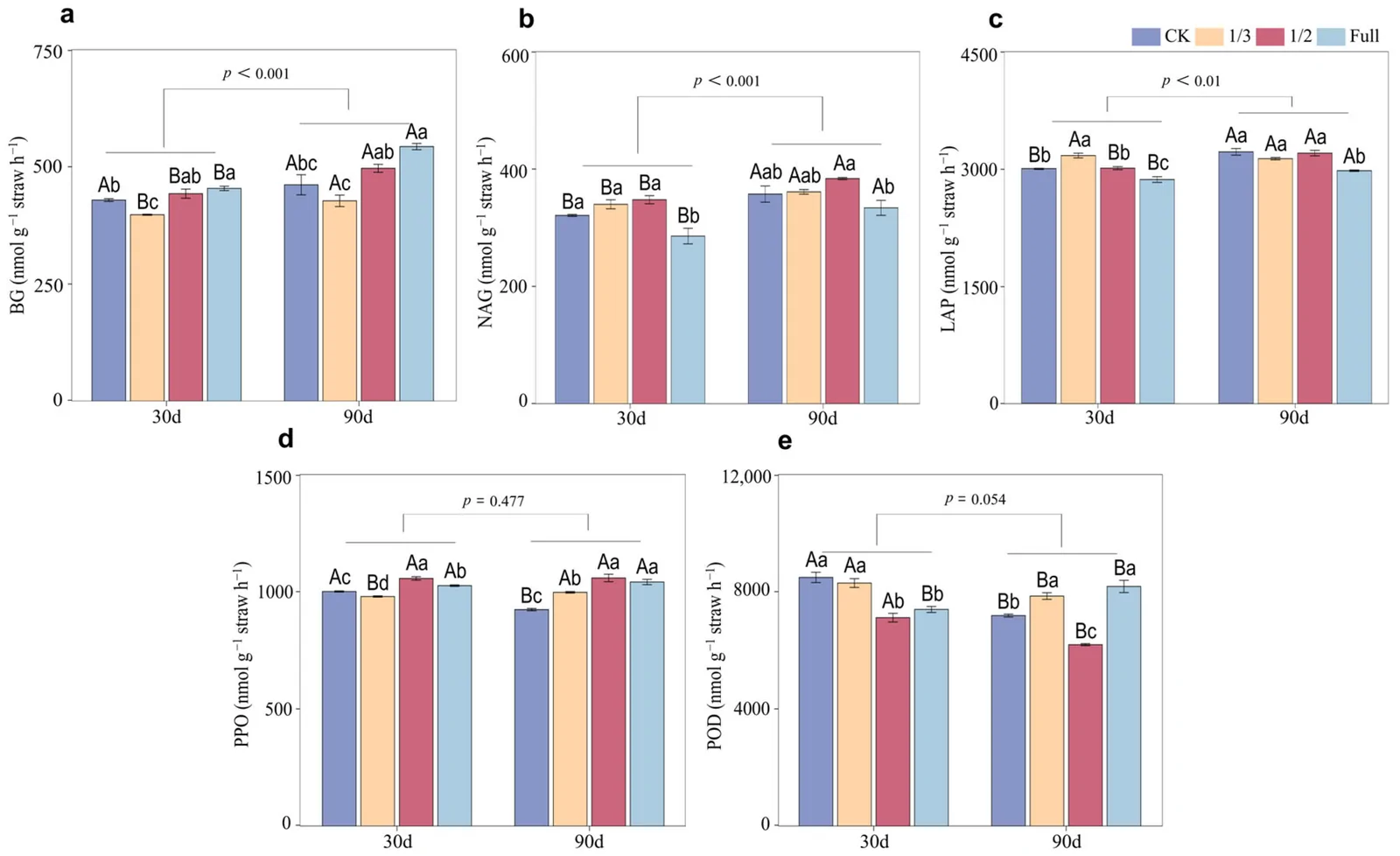
Activities of five extracellular enzymes in Mollisols with different straw return rate at 30 d and 90 d. (**a**) β-glucosidase (BG); (**b**) N-acetyl-β-D-glucosaminidase (NAG); (**c**) leucine aminopeptidase (LAP); (**d**) polyphenol oxidase (PPO); (**e**) peroxidase (POD). Error bars indicate SE. Within each sampling time point, different lowercase letters above columns indicate significant differences among treatments (Tukey’s HSD test, *p* < 0.05); within the same straw return treatment, different uppercase letters denote significant differences between the two sampling times (Student’s *t*-test, *p* < 0.05). Data are based on four replicates (*n* = 4).

The five extracellular enzymes showed distinct temporal patterns during straw decomposition (Figure 3). The activities of the three hydrolytic enzymes increased from 30 to 90 d, including BG (*p* < 0.001), NAG (*p* < 0.001) and LAP (*p* < 0.01). In contrast, PPO showed no significant temporal change (*p* = 0.477), while POD exhibited a marginal decline (*p* = 0.054). Return rate also influenced enzyme activity within each sampling time, with BG generally increasing with straw return rate and reaching the highest level under the Full treatment at both stages. NAG showed its lowest activity under the Full treatment at 30 d, whereas the other enzymes displayed treatment-specific patterns.

Correlation analysis further showed that hydrolytic enzymes, particularly BG, were positively associated with straw mass loss, whereas PPO and POD were weakly or negatively related to decomposition (Figure S6). These results indicate that hydrolytic enzyme activity was more closely linked to straw breakdown than oxidative activity in this system.

### 3.5. Reprogramming of the Straw Metabolome

**Figure 4:**
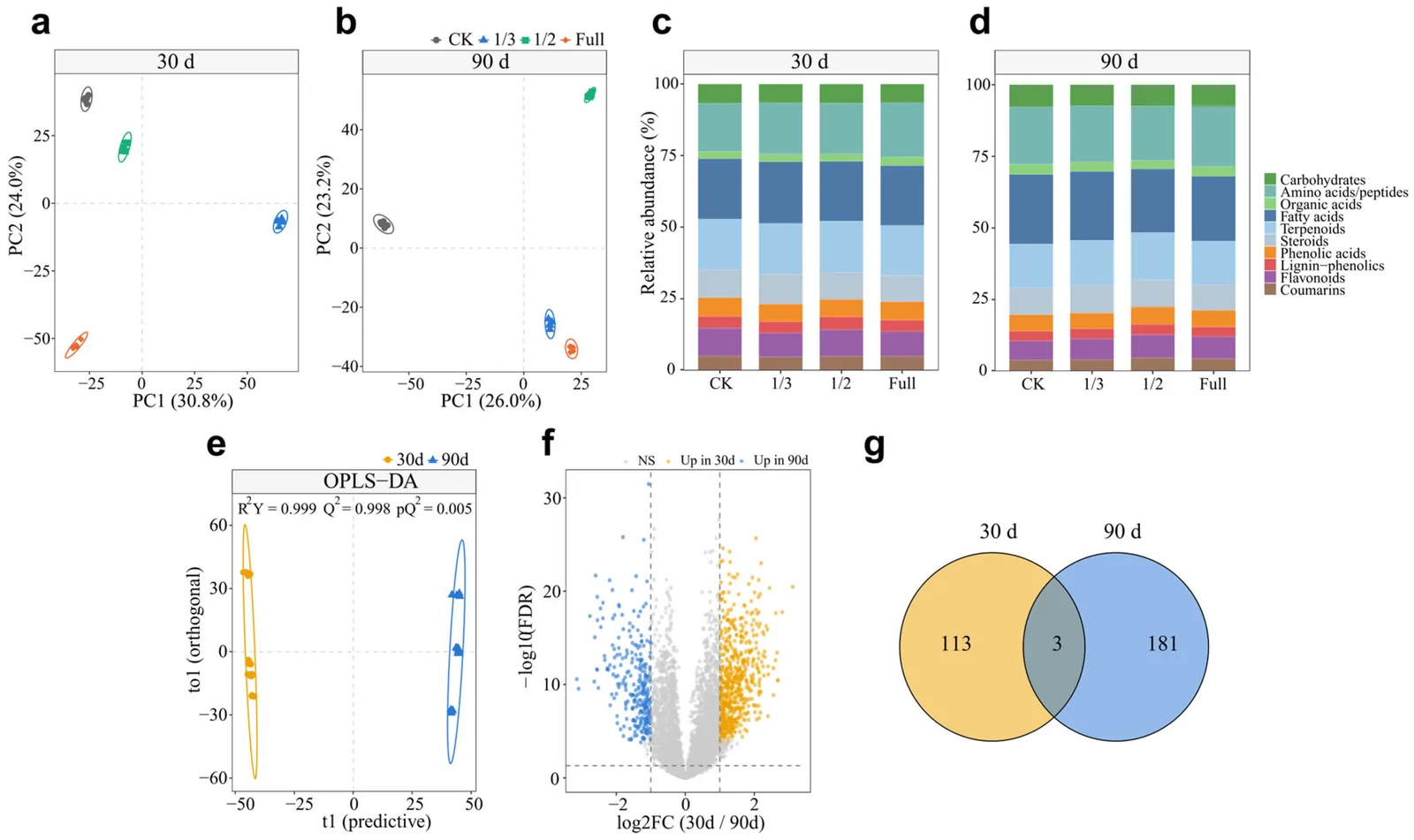
Differential metabolome profiles and functional composition of straw metabolites across treatments and stages. PCA score plots at 30 d (**a**) and 90 d (**b**) across treatments; Relative abundance of functional groups at 30 d (**c**) and 90 d (**d**); (**e**) OPLS-DA score plot distinguishing 30 d and 90 d; (**f**) Volcano plot of DEMs; (**g**) Venn diagram of up-regulated metabolites at two stages showing minimal overlap (3 shared metabolites).

Untargeted metabolomics revealed strong temporal and treatment-related reprogramming of the straw metabolome (Figure 4a–g and Figure S3). PCA separated samples by both straw return treatment and incubation time (Figure 4a,b), and OPLS-DA clearly discriminated the 30 d and 90 d metabolomes (Figure 4e).

The functional composition of differentially expressed metabolites changed substantially between stages (Figure 4c,d,f). Metabolites up-regulated at 30 d were mainly assigned to labile compound classes, including carbohydrates, amino acids and peptides, organic acids and fatty acids. By contrast, metabolites up-regulated at 90 d were enriched in aromatic and phenolic classes, including lignin-related phenolics, phenolic acids, flavonoids and coumarins. The two stage-specific metabolite sets showed almost no overlap, indicating a near-complete turnover of the straw metabolome during decomposition. Consistently, only three metabolites were shared between the sets enriched under straw return relative to CK at 30 d and 90 d (Figure 4g).

KEGG pathway enrichment analysis further supported distinct functional shifts between stages (Figure S4). Together, these results indicate a transition from a metabolome dominated by labile compounds at 30 d to one enriched in aromatic and phenolic metabolites at 90 d.

### 3.6. Multi-Omics Coupling Among Metabolomes, Microbiomes and Enzymes

**Figure 5:**
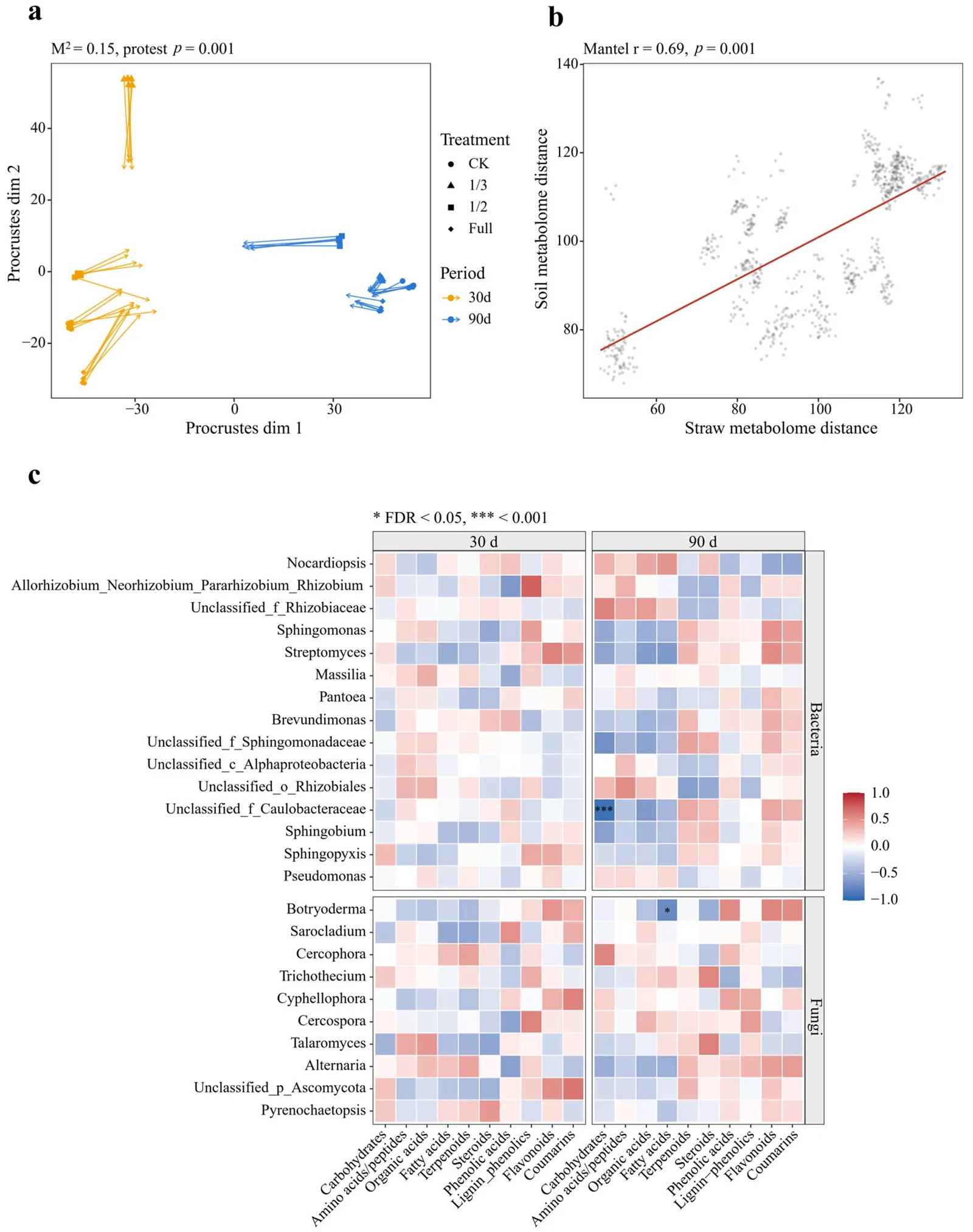
Multi-omics integration revealing metabolome coupling and microbe-metabolite associations. (**a**) Procrustes analysis of straw vs. soil metabolomes (M^2^ = 0.15, protest *p* = 0.001). (**b**) Mantel test showing the correlation between straw and soil metabolome distances (r = 0.69, *p* = 0.001). (**c**) Spearman correlation heatmap between dominant microbial genera and metabolite functional groups at 30 d and 90 d. Asterisks denote FDR-corrected significance (* *p* < 0.05, *** *p* < 0.001).

The straw and soil metabolomes were tightly coupled across treatments and sampling times, as shown by Procrustes analysis (M^2^ = 0.15, *p* = 0.001) and Mantel analysis (Figure 5a,b). This indicates that changes in the straw metabolite pool were closely associated with changes in the surrounding soil metabolome.

Stage-resolved correlation analysis revealed distinct associations between dominant microbial genera and metabolite functional groups at 30 d and 90 d (Figure 5c). Similarly, the integrated network linking microbial genera, metabolite groups, enzyme activities and decomposition rate showed stage-dependent co-occurrence patterns (Figure S5). In addition, Mantel network analysis identified significant stage-specific links between environmental factors and the straw metabolome, as well as between environmental factors and fungal community composition at 90 d (Figure S7). Together, these analyses demonstrate a temporally dynamic coupling among metabolite profiles, microbial communities and enzyme activities during straw decomposition.

### 3.7. Microbial Drivers of Straw Decomposition

**Figure 6:**
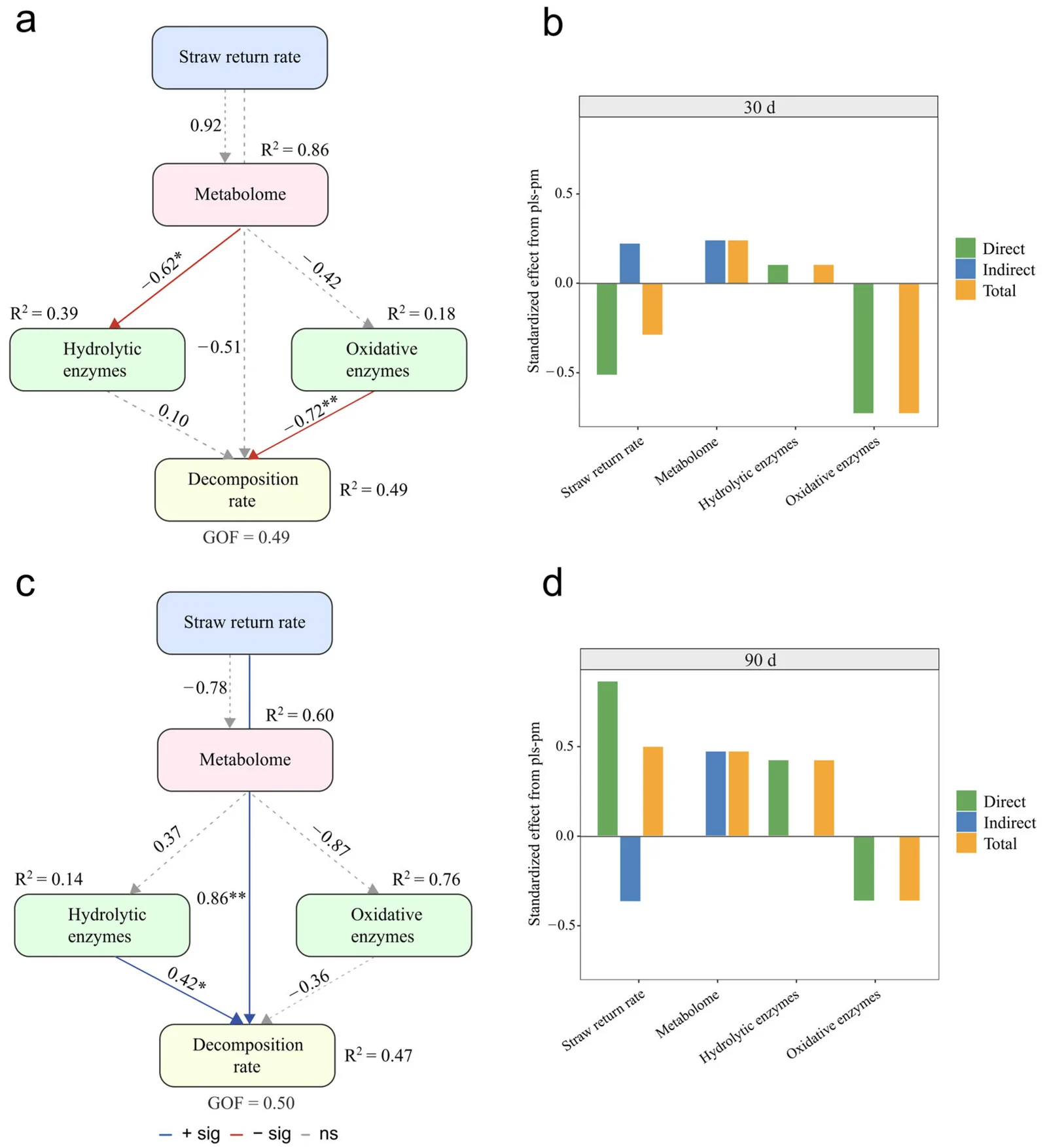
Partial least squares path modeling (PLS-PM) analysis of drivers for straw decomposition. Panels (**a**,**c**) show the path models at 30 and 90 days post-incorporation, respectively, displaying standardized path coefficients and variable R^2^ values. Solid blue and red arrows denote significant positive and negative effects (* *p* < 0.05, ** *p* < 0.01,), respectively; gray dashed arrows indicate non-significant (ns) effects. GOF indicates the goodness-of-fit of the model. Panels (**b**,**d**) show the standardized direct (green), indirect (blue), and total (orange) effects of each factor on the decomposition rate at 30 and 90 days, respectively.

PLS-PM was used to disentangle the direct and indirect effects of straw return rate on decomposition at each sampling time (Figure 6). At 30 d, the relationships among return rate, metabolome and microbial community were relatively weak, and decomposition was more strongly associated with enzyme-mediated pathways. The variation in decomposition was explained by a combination of direct and indirect influences, with oxidative enzymes showing a negative association with decomposition and metabolite-related pathways contributing positively.

At 90 d, the model structure changed substantially. Straw return rate exerted a strong direct positive effect on decomposition, while also reshaping the metabolome and enzyme activities. Hydrolytic enzyme activity was positively associated with decomposition, whereas oxidative enzyme activity remained negatively related. The relative importance of direct and indirect pathways therefore shifted between 30 d and 90 d, indicating a stage-dependent change in the mechanisms governing straw mass loss. Overall, the PLS-PM results show that early decomposition was primarily linked to enzymatic mediation, whereas later decomposition was more strongly influenced by straw return rate and the associated restructuring of the metabolome and enzyme system.

## 4. Discussion

### 4.1. Straw Return Rate Significantly Affected Straw Decomposition and Soil Carbon and Nitrogen Dynamics

Straw decomposition increased markedly from 30 to 90 d, while the effect of straw return rate became evident only at 90 d. At 30 d, straw mass loss ranged from approximately 21% to 25% and did not differ significantly among treatments. By 90 d, mass loss was significantly higher, and it responded nonlinearly to straw return rate: mass loss peaked at intermediate rather than full straw return (Figure 1a). This nonlinear pattern suggests that factors beyond substrate quantity (such as microbial C use efficiency, nutrient availability, or physical protection within residue layers) may constrain decomposition when straw input is high [11,21]. Notably, greater residue mass loss did not translate into greater short-term SOC accumulation. At 90 d, SOC content and SOC increment did not increase monotonically with return rate, and the treatment associated with the highest SOC and TN contents did not correspond to the treatment with the greatest mass loss (Figure S1a–c). These results indicate a decoupling between straw mass loss and short-term soil C and N retention, which should be interpreted with caution. Straw mass loss reflects residue disappearance, whereas SOC represents the net outcome of residue inputs, microbial transformation, mineralization, and other soil processes [9,33]. Since CO_2_ release, microbial biomass C, and different SOC fractions were not measured in this study, the mechanisms underlying the SOC differences cannot be resolved directly.

Soil C/N ratio increased from 30 to 90 d across all treatments, but straw return rate had no significant effect on soil C/N at either sampling time (Figure S1d). In contrast, straw C and N contents declined during incubation, and straw C/N decreased with increasing return rate at 30 d (Figure 1b–d). Together, these results indicate that straw return rate mainly influenced residue decomposition and soil C and N accumulation in a time-dependent manner, whereas its effect on soil C/N stoichiometry was relatively limited. Overall, straw return rate shaped decomposition dynamics and soil C and N responses in a stage-dependent fashion, and the dissociation between mass loss and soil C accumulation underscores the need for a mechanistic, rather than input-output, understanding of residue decomposition in Mollisols.

### 4.2. Stage-Specific Metabolite Turnover During Straw Decomposition

The straw metabolome differed markedly between 30 and 90 d and also varied among straw return treatments (Figure 4a–g; Figure S3). At 30 d, the metabolite profile was dominated by carbohydrates, amino acids and peptides, organic acids, and fatty acids, whereas at 90 d it shifted toward aromatic and phenolic compounds, including lignin-related phenolics, phenolic acids, flavonoids, and coumarins (Figure 4c,d,f). The limited overlap between the stage-specific metabolite sets suggests substantial turnover of straw metabolites during incubation.

This shift indicates that the chemical composition of the remaining straw changed substantially over time, with relatively labile compounds being more prominent at the early stage and aromatic/phenolic compounds becoming more abundant at the later stage [7,34]. However, these metabolomic results reflect compound composition rather than complete degradation pathways; they do not directly demonstrate that all labile compounds were preferentially mineralized or that aromatic compounds necessarily accumulated solely because of recalcitrance. Other processes, such as microbial necromass accumulation and de novo synthesis of secondary metabolites, may also contribute to the observed shift [10].

The clear separation between the 30- and 90 d metabolomes in multivariate analyses further confirms that decomposition stage was a major driver of straw chemical change. In addition, the significant coupling between straw and soil metabolomes suggests coordinated chemical changes in the residue-soil system, although it does not reveal the direction of metabolite exchange or causality. These stage-specific metabolite profiles provide the chemical context in which microbial metabolism and enzyme production operate during straw decomposition.

### 4.3. Stage-Dependent Succession of Microbial Communities

Both bacterial and fungal community compositions differed among straw return treatments and between sampling times (Figure 2a,b,d,e). PERMANOVA showed significant compositional differences, while PERMDISP was non-significant for most comparisons, indicating that the observed separation mainly reflected community composition rather than differences in dispersion. The only exception was bacteria at 30 d, where significant dispersion heterogeneity suggests that this early-stage separation should be interpreted cautiously.

Bacterial communities showed a clear temporal shift in taxonomic composition, with increased relative abundance of Alphaproteobacteria at 90 d and decreased abundance of Actinobacteriota and other early-stage taxa (Figure 2c). Fungal communities were consistently dominated by Sordariomycetes, although the relative abundances of Eurotiomycetes, Agaricomycetes, and unclassified Ascomycota changed over time (Figure 2f). These patterns indicate microbial succession during straw decomposition, without supporting a simple transition from bacterial to fungal dominance [8,35].

Differential-abundance analysis showed that the taxa responding to straw return varied by decomposition stage. At 30 d, Massilia was enriched among bacteria, while Talaromyces and Cladosporium were enriched among fungi. At 90 d, Pantoea, Tardiphaga, and Lysinimonas were enriched among bacteria, whereas Cercospora was enriched among fungi (Figure 2g,h). This stage dependence suggests that different microbial taxa were favored as residue chemistry changed over the course of decomposition. However, taxonomic enrichment alone does not reveal the functional roles of these taxa in decomposition.

Alpha diversity also changed with incubation time and straw return treatment (Figure S2). Together with the ordination results, these patterns show that microbial communities were reshaped throughout decomposition. The observed associations between microbial genera and metabolite groups further suggest that community turnover occurred alongside changes in residue chemistry, providing the biological basis for the stage-specific metabolic and enzymatic responses that drive straw decomposition [36,37,38].

### 4.4. Hydrolytic Enzymes as the Primary Drivers of Straw Mass Loss

Hydrolytic and oxidative enzymes responded differently during straw decomposition (Figure 3). BG, NAG, and LAP activities increased significantly from 30 to 90 d, whereas PPO showed no significant change and POD declined slightly. These patterns indicate that enzyme activity was strongly stage dependent.

Among the measured enzymes, hydrolytic enzymes (especially BG) were more closely associated with straw mass loss than oxidative enzymes (Figure S6). The positive correlations between BG, NAG, LAP, and decomposition suggest that hydrolytic activity was more directly linked to residue mass loss in this experiment, whereas PPO and POD showed weak or negative relationships with decomposition. Notably, enzyme activity did not scale linearly with return rate: BG activity generally increased with greater straw input, but the highest enzyme activity did not coincide with the greatest mass loss. This implies that enzyme activity alone is insufficient to explain decomposition outcomes; substrate chemistry, physical accessibility, and microbial community composition likely modulate the relationship between enzyme production and residue breakdown [39].

Individual enzymes also responded differently to straw return. BG generally increased with increasing straw return rate, while NAG showed a different pattern, particularly at 30 d (Figure 3). These results suggest that increasing straw input did not trigger a uniform enzymatic response across hydrolytic and oxidative pathways. The differential responses of hydrolytic and oxidative enzymes to both stage and return rate highlight the functional complexity of extracellular enzyme networks during decomposition [40,41].

PLS-PM further indicated that the relationships among straw return rate, metabolites, microbial communities, enzymes, and decomposition changed over time (Figure 6). At 30 d, decomposition was more closely linked to enzyme-related pathways, whereas at 90 d, straw return rate and hydrolytic enzymes showed stronger associations with mass loss. Overall, these results suggest that hydrolytic enzymes were more directly related to straw decomposition than oxidative enzymes, but the relationship between enzyme production and decomposition is mediated by substrate chemistry and community composition in a stage-dependent manner, rather than being a simple function of enzyme abundance.

### 4.5. Integrated Interpretation of Stage-Specific Decomposition Mechanisms

The results support a stage-dependent framework in which straw return rate modulates decomposition through coupled changes in residue chemistry, microbial community structure, and extracellular enzyme activity. At 30 d, decomposition was slow and did not differ significantly among treatments, while the straw metabolome was dominated by relatively labile compounds and microbial communities showed early-stage treatment responses (Figure 1a, Figure 2 and Figure 4). PLS-PM indicated that decomposition at this stage was more closely associated with enzyme-related pathways than with the direct effect of straw return rate (Figure 6), suggesting that early-stage decomposition was driven primarily by the metabolic activity of the initial microbial community rather than by substrate quantity.

By 90 d, the residual straw was enriched in aromatic and phenolic metabolites, microbial communities had undergone clear compositional turnover, and hydrolytic enzyme activities were substantially higher than at 30 d (Figure 2, Figure 3 and Figure 4). Decomposition differed significantly among treatments, and the PLS-PM model showed stronger associations among straw return rate, metabolome, enzyme activities, and decomposition at this stage (Figure 6). This temporal shift suggests that the relative importance of return rate, substrate chemistry, and enzyme activity in driving decomposition was reorganized as decomposition progressed. Specifically, the microbial metabolic network driving decomposition is itself reshaped by the changing chemical environment of the decomposing residue.

The integrated correlation analyses further revealed stage-specific links between the metabolome, environmental factors, and microbial community composition (Figure 5; Figure S7). However, these correlations indicate coordinated variation rather than causal relationships, and the specific microbial metabolic pathways connecting residue chemistry to decomposition remain to be resolved. Furthermore, greater residue mass loss did not necessarily correspond to greater short-term SOC and TN accumulation (Figure 1a; Figure S1a–c), underscoring that decomposition rate and soil C retention are decoupled processes. Overall, straw decomposition in this Mollisol system was driven by stage-specific coupling among residue chemistry, microbial community composition, and enzyme activity, with their relative contributions shifting over the incubation period.

### 4.6. Limitations and Future Research

Several limitations should be acknowledged. First, the 90-day incubation captures only the early-to-middle stages of straw decomposition. Therefore, the long-term fate of residue-derived C remains unclear. In particular, it is uncertain whether the short-term SOC differences observed in this study persist, stabilize, or reverse over multi-season time scales. Second, sampling at only two time points (30 and 90 d) limits the temporal resolution of microbial succession and metabolite turnover. Third, CO_2_ efflux, microbial biomass C, and SOC fractions (e.g., particulate and mineral-associated organic C) were not measured. This prevents us from directly resolving the mechanisms underlying the observed SOC differences. Future studies should extend observations across multiple growing seasons and freeze–thaw cycles. They should also increase sampling frequency, quantify CO_2_ emissions and C fractions, and apply isotope tracing (^13^C) to track residue C from decomposition to stabilization.

## 5. Conclusions

Straw return rate shaped straw decomposition through stage-dependent coupling among microbial communities, metabolites, and enzyme activities. Straw mass loss responded nonlinearly to return rate, increasing from 21–25% at 30 d to a maximum of 53.7% under the 1/2 return treatment at 90 d. Residue mass loss was decoupled from short-term soil C and N accumulation: at 90 d, SOC and TN were highest under the 1/3 return treatment, exceeding the control by 17.6% and 17.2%, respectively, rather than under the treatment with the greatest mass loss. This indicates that the factors driving decomposition differ from those governing soil C and N retention. The straw metabolome shifted from labile compounds at 30 d to aromatic and phenolic compounds at 90 d, and straw and soil metabolomes were tightly coupled (Procrustes M^2^ = 0.15; Mantel r = 0.69), accompanied by temporal changes in microbial community composition and enzyme activity. Hydrolytic enzymes were more closely associated with mass loss than oxidative enzymes, and the relationships among return rate, metabolome, enzymes, and decomposition were reorganized between stages. Overall, these findings demonstrate that microbial communities drive straw decomposition through stage-specific metabolic and enzymatic responses and that residue breakdown and soil C accumulation are nonlinear, decoupled processes that are best understood through their underlying mechanisms.

## Data Availability

The data that support the findings of this study are available from the corresponding author upon reasonable request. The data are not publicly available due to privacy restrictions.

## Supplementary Materials

The following supporting information can be downloaded at https://www.mdpi.com/article/10.3390/microorganisms14091929/s1: Figure S1. Soil organic carbon (SOC) content (**a**), SOC increment (**b**), total nitrogen (TN) content (**c**), and soil C:N ratio (**d**) in Mollisols under different straw return rates at 30 and 90 days post-incorporation. Figure S2. Bacterial Shannon index (**a**), fungal Shannon index (**b**), bacterial Simpson index (**c**), and fungal Simpson index (**d**) in Mollisols under different straw return rates at 30 and 90 days post-incorporation. Figure S3. Straw metabolome profiles and functional group changes under different return rates. Figure S4. KEGG pathway enrichment analysis of straw DEMs enriched at 30 d and 90 d. Figure S5. Spearman correlation networks showing the co-occurrence patterns among microbial genera, metabolite functional groups, enzyme activities, and decomposition rate at 30 d (**a**) and 90 d (**b**). Figure S6. Linear correlations between five extracellular enzyme activities and straw decomposition rate. Figure S7. Mantel test network showing links between environmental factors and omics datasets at 30 d (**a**) and 90 d (**b**).; Table S1. Parameters for five enzyme assays. Table S2. Relative abundance of dominant bacterial classes under different straw return rates at 30 and 90 days; Table S3. Relative abundance of dominant fungal classes under different straw return rates at 30 and 90 days; Table S4. Results of two-way PERMANOVA testing the effects of straw return rate and sampling period on bacterial and fungal beta diversity.
