# Supplementary material for: Microbes Drive Straw Decomposition and Microbial Metabolism in Mollisols with Different Straw Return Rates
Source: Microorganisms. 2026 Sep 1;14(9):1929. doi: 10.3390/microorganisms14091929 (PMC13609777; doi:10.3390/microorganisms14091929)
Supplement: Supplementary file 1 [file microorganisms-14-01929-s001.zip › Figure.pdf]

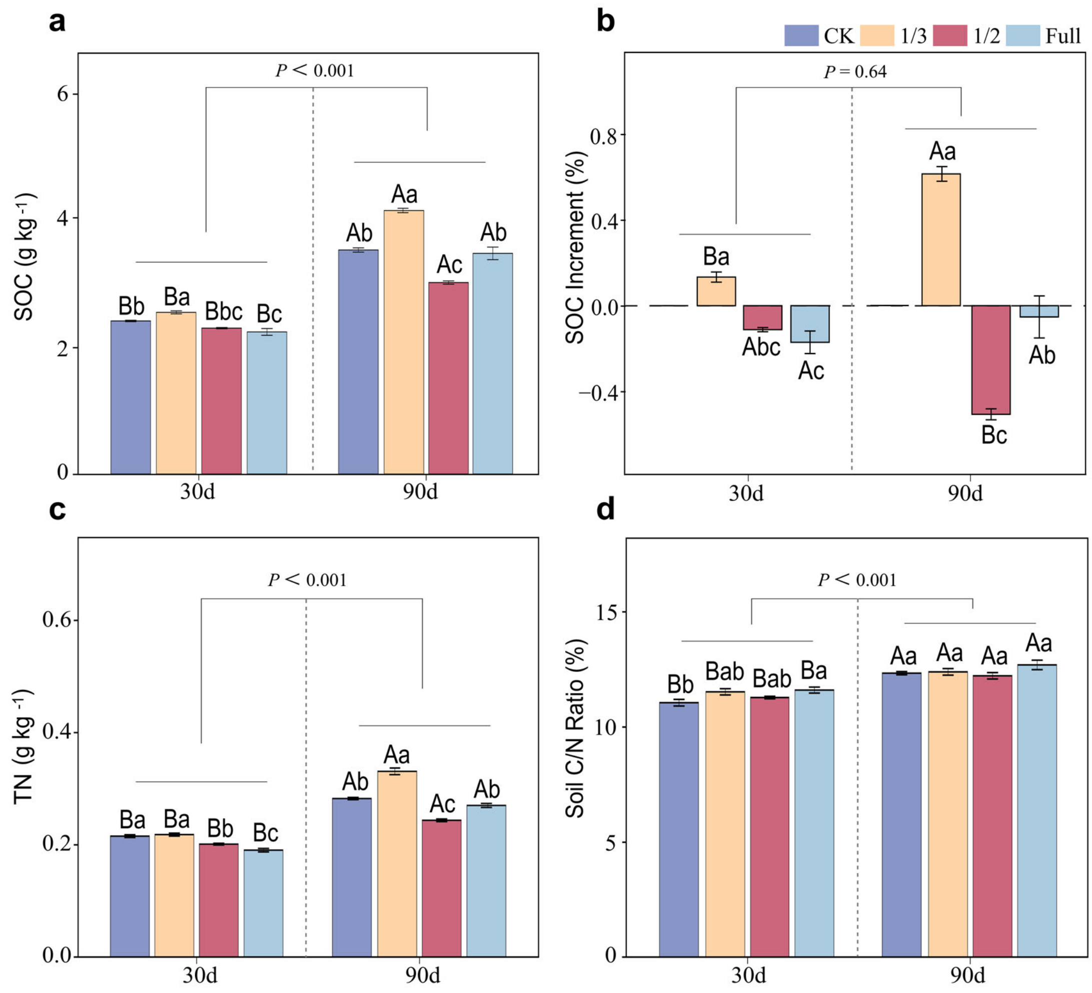

Figure S1. Soil organic carbon (SOC) content (**a**), SOC increment (**b**), total nitrogen (TN) content (**c**), and soil C:N ratio (**d**) in Mollisols under different straw return rates at 30 and 90 days post-incorporation. The treatments CK, 1/3, 1/2, and Full represent the control (no straw return), one-third, half, and full straw return rates, respectively. Bars represent means ± standard error (n = 4). Within each sampling time point (30 d or 90 d), different lowercase letters above the columns indicate significant differences among treatments (Tukey's HSD test, $P < 0.05$); within the same straw incorporation treatment, different uppercase letters denote significant differences between the two sampling times (Student's t -test, $P < 0.05$). No letters are shown for CK in panel (**b**), as the SOC increment is defined relative to CK and is therefore invariably zero.

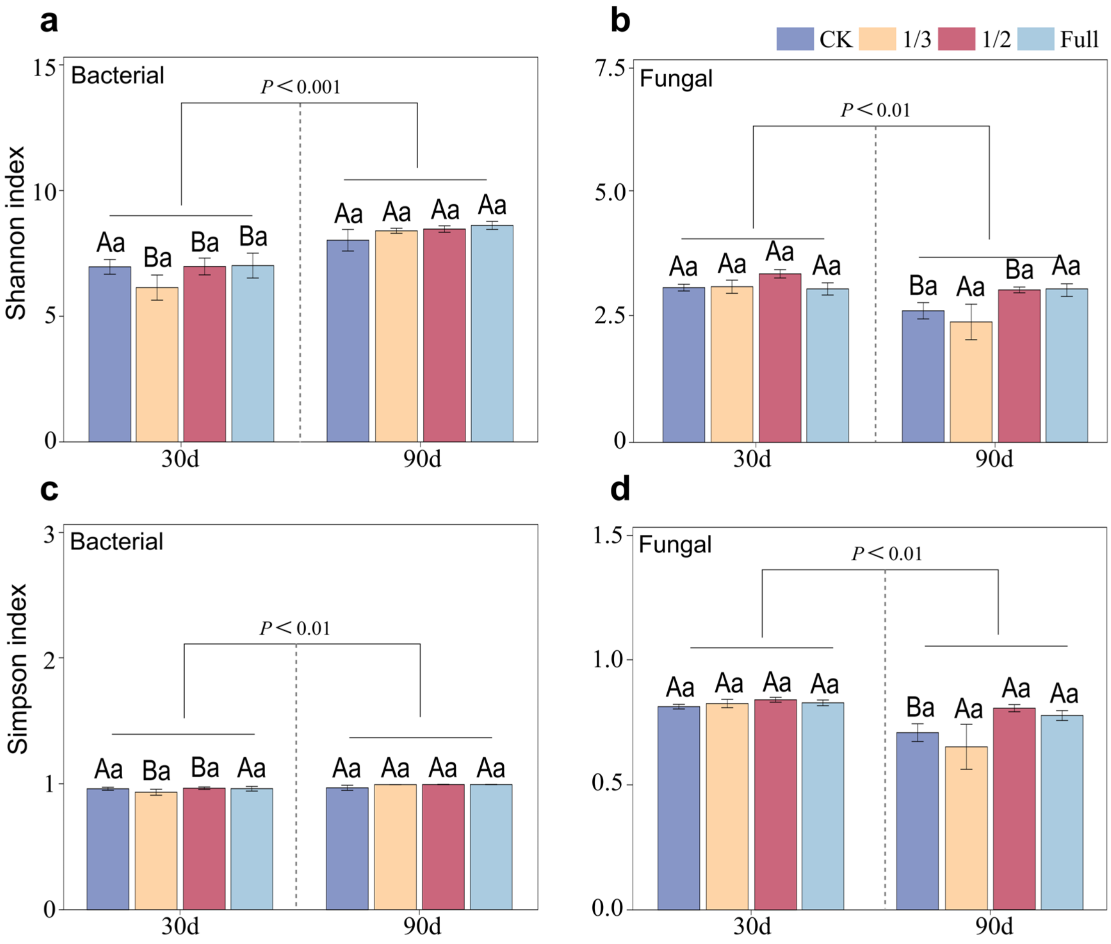

Figure S2. Bacterial Shannon index (**a**), fungal Shannon index (**b**), bacterial Simpson index (**c**), and fungal Simpson index (**d**) in Mollisols under different straw return rates at 30 and 90 days post-incorporation. The treatments CK, 1/3, 1/2, and Full represent the control (no straw return), one-third, half, and full straw return rates, respectively. Bars represent means ± standard error (n = 4). Within each sampling time point, lowercase letters above columns indicate significant differences among treatments (Tukey's HSD test, $P < 0.05$); within the same straw incorporation treatment, uppercase letters denote significant differences between the two sampling times (Student's t-test, $P < 0.05$). Columns sharing a letter are not significantly different. Overarching brackets with *P*-values indicate overall significant time effects.

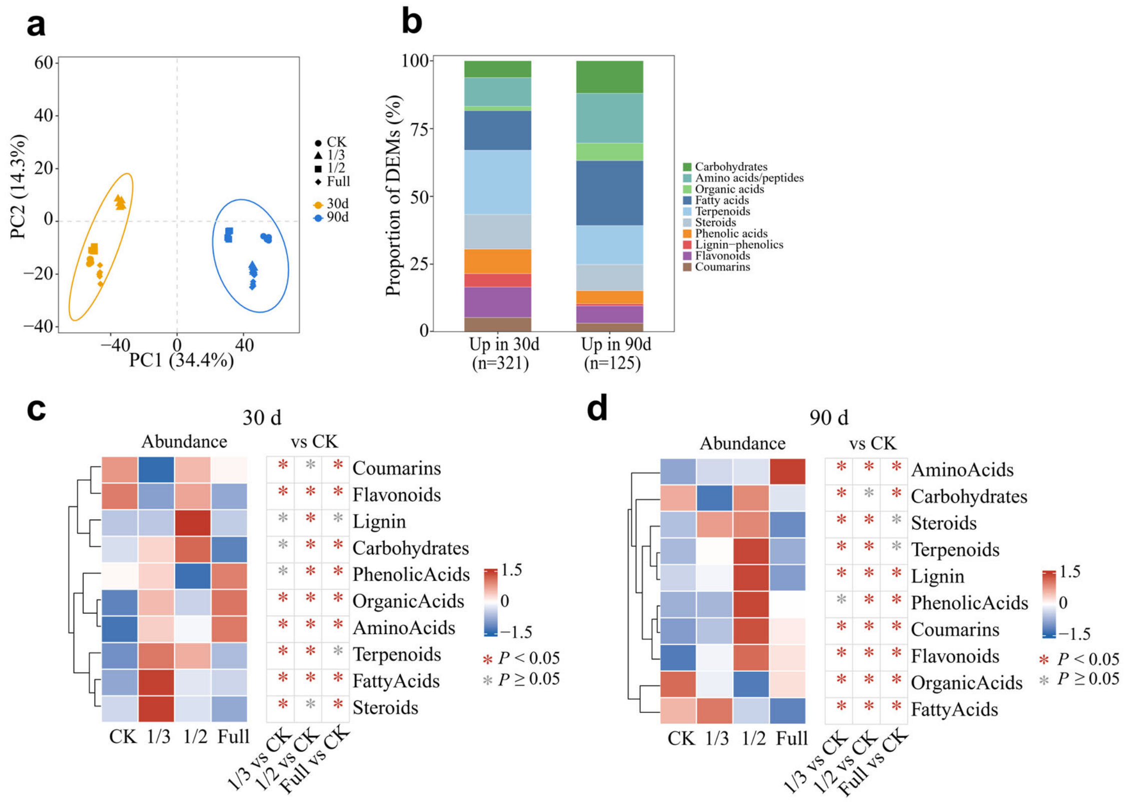

Figure S3. Straw metabolome profiles and functional group changes under different return rates. (**a**) PCA score plot of straw metabolome at 30 d and 90 d; (**b**) Relative proportion of functional groups in DEMs enriched at 30 d (n=321) and 90 d (n=125); Heatmaps of functional group abundance and their statistical contrasts vs. CK at 30 d (**c**) and 90 d (**d**). Asterisks indicate significant differences vs. CK ($P < 0.05$).

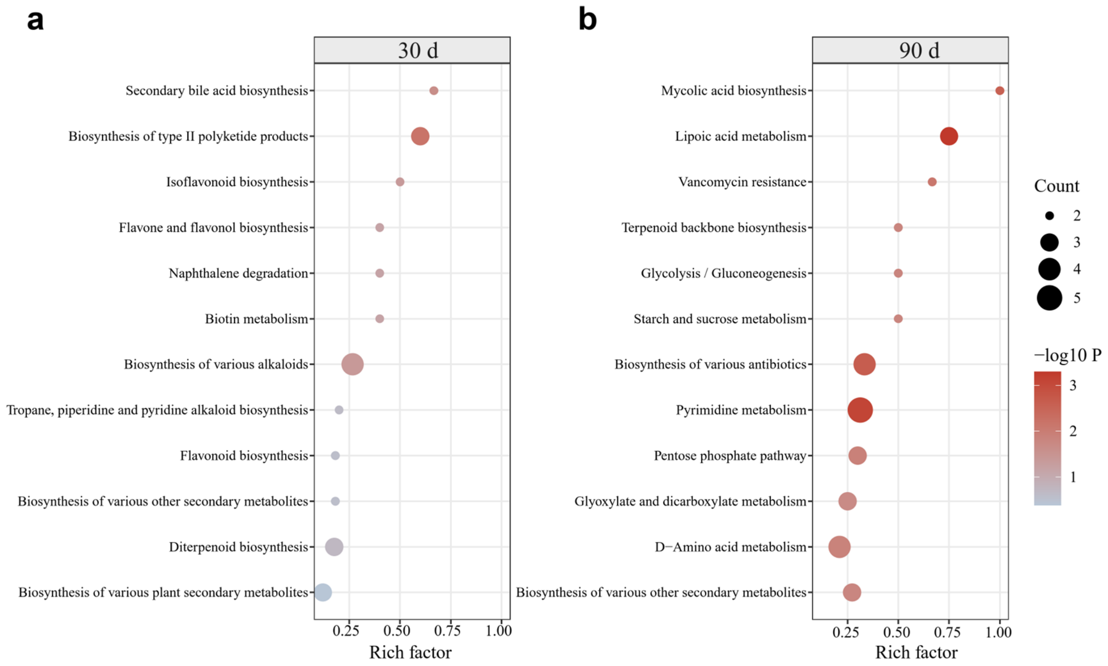

Figure S4. KEGG pathway enrichment analysis of straw DEMs enriched at 30 d and 90 d. (**a**) Significantly enriched pathways at 30 d; (**b**) significantly enriched pathways at 90 d. The x-axis indicates the rich factor (the ratio of DEMs to total annotated metabolites within each pathway). Bubble size (Count) represents the number of enriched metabolites, and bubble color represents the statistical significance (-log10 *P*-value; darker red denotes lower *P*-values). Note: Putative pathway enrichment based on metabolite annotations.

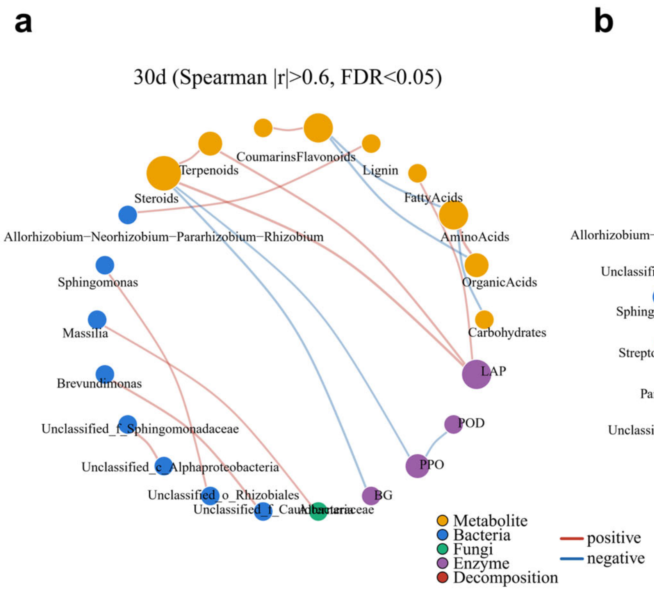

Figure S5. Spearman correlation networks showing the co-occurrence patterns among microbial genera, metabolite functional groups, enzyme activities, and decomposition rate at 30 d (**a**) and 90 d (**b**). Only significant correlations with |r| > 0.6 and FDR < 0.05 are shown. Red and blue edges indicate positive and negative correlations, respectively. Node sizes are proportional to node degree.

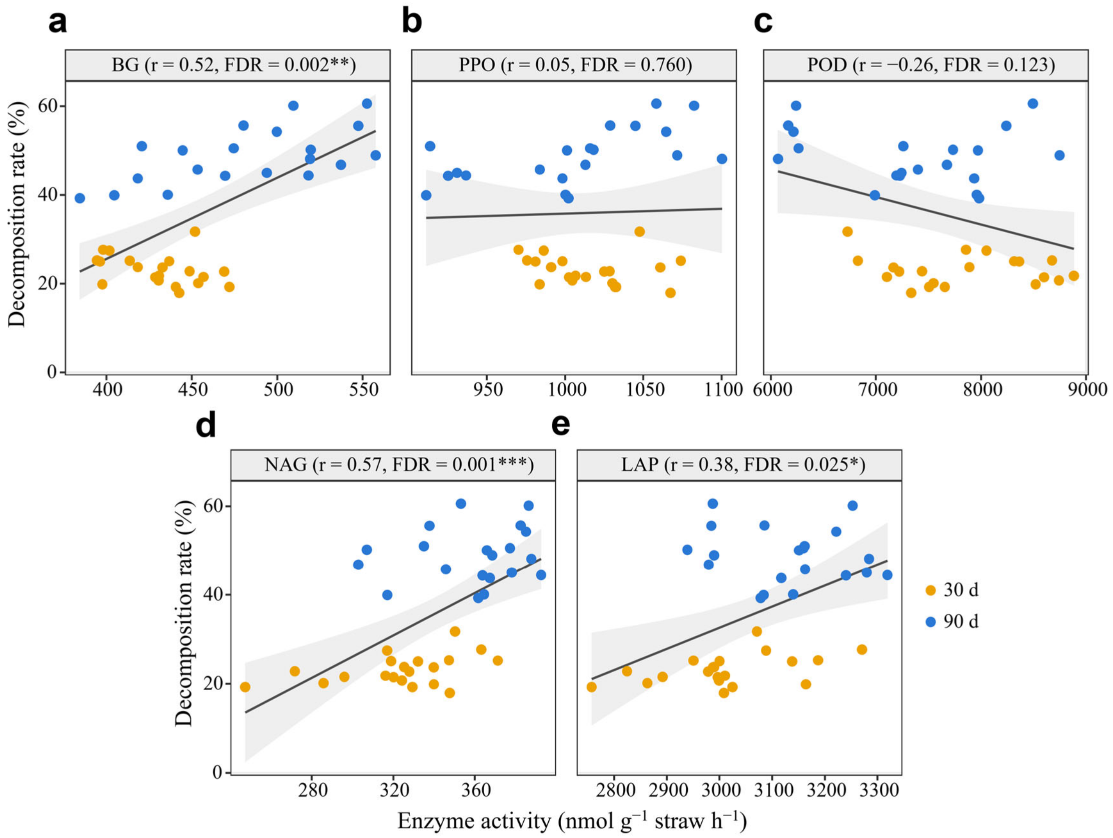

Figure S6. Linear correlations between five extracellular enzyme activities and straw decomposition rate. Scatter plots showing the relationships between decomposition rate and the activities of BG (**a**), PPO (**b**), POD (**c**), NAG (**d**), and LAP (**e**). Yellow and blue dots denote samples at 30 d and 90 d, respectively. Spearman correlation coefficients (r) and FDR-corrected significance levels are shown in each panel. Shaded areas represent 95% confidence intervals of the linear regressions.

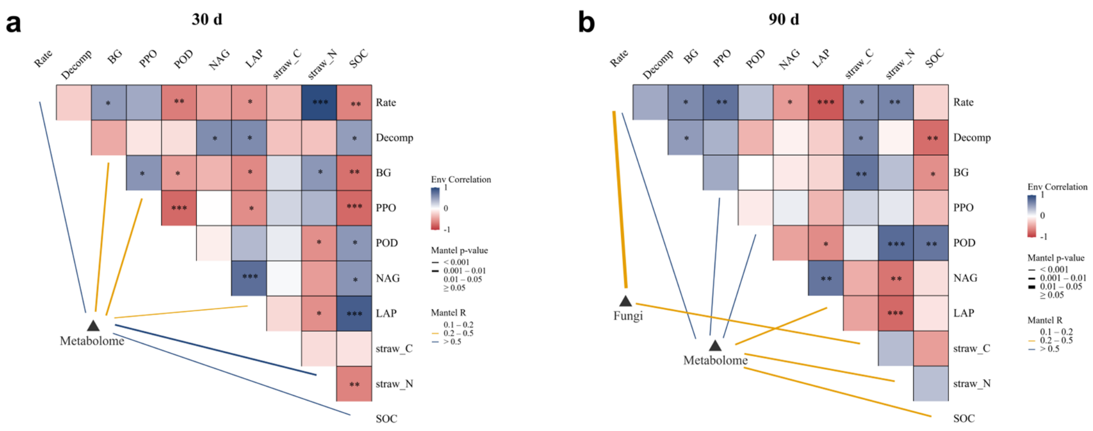

Figure S7. Mantel test network showing links between environmental factors and omics datasets at 30 d (**a**) and 90 d (**b**). The lower-left triangular heatmap displays Spearman correlations among environmental variables; asterisks indicate significance. Curves represent Mantel tests between environment and metabolome (and fungi at 90 d). Curve color denotes Mantel r, while line type indicates FDR-corrected significance (solid: FDR < 0.01; dashed: FDR < 0.05). Bacterial connections were omitted due to non-significant FDR-corrected constraints.
