# Supplementary material for: Microbes Drive Straw Decomposition and Microbial Metabolism in Mollisols with Different Straw Return Rates
Source: Microorganisms. 2026 Sep 1;14(9):1929. doi: 10.3390/microorganisms14091929 (PMC13609777; doi:10.3390/microorganisms14091929)
Supplement: Supplementary file 1 [file microorganisms-14-01929-s001.zip › Table.pdf]

Table S1. Parameters for five enzyme assays.

Enzyme	Assay method	Detection wavelength (nm)	Extraction volume V (mL)	Sample loading volume V1 (mL)	Total reaction volume V2 (mL)	Incubation time T (min)	Dilution factor (DF)	Sample weight W (g)	Standard curve	Unit definition and calculation formula
PPO (PO)	Dopachrome method	490	1	0.1	0.2	30	1	0.1	1	PO (ΔOD490min/g) = $(A2-A1) / (W/V*V1) / 0.001 / T*DF$
NAG	p-Nitrophenol colorimetric method	400	1	0.01	0.07	30	5	0.1	5	NAG (nmol/min/g) = $[(\Delta A - 0.0083)/0.0039 * V2] / (V1/V * W) / T * DF$
BG (B-GC)	p-Nitrophenol colorimetric method	400	1	0.03	0.3	30	5	0.1	5	BG (nmol/min/g) = $[(\Delta A + 0.0027)/0.0039 * V2] / (V1/V * W) / T * DF$
POD	Guaiacol method	470	1	0.01	0.2	1	1	0.1	1	POD (U/g) = $(A2-A1) * V2/ (V1/V*W) / 0.005 / T*DF$
LAP	p-Nitroaniline colorimetric method	405	1	0.05	0.2	2	5	0.1	5	LAP (nmol/min/g) = $[(A2 - A1) * V2] / (\in * d) * 10^9 / (W/V * V1)/T * DF)$

Note: BG, β-glucosidase; NAG, N-acetyl-β-D-glucosaminidase; LAP, leucine aminopeptidase; PPO, polyphenol oxidase; POD, peroxidase. The calculation formulas for PPO and POD were based on the direct absorbance change, while BG, NAG, and LAP were based on standard curves or extinction coefficients.

Table S2. Relative abundance of dominant bacterial classes under different straw return rates at 30 and 90 days.

Time	Class	CK	1/3	1/2	Full
30d	Alphaproteobacteria	29.8 ± 4 a	38.1 ± 3.6 a	38.9 ± 3.9 a	37.9 ± 5.6 a
	Acidobacteriae	0 ± 0 a	0.1 ± 0 a	0.2 ± 0.2 a	0 ± 0 a
	Verrucomicrobiae	0 ± 0 a	0 ± 0 a	0 ± 0 a	0 ± 0 a
	Gemmatimonadetes	1.1 ± 0.3 a	1.1 ± 0.1 a	0.9 ± 0.2 a	0.8 ± 0.3 a
	Gammaproteobacteria	17.8 ± 4.9 a	33.9 ± 6.1 a	28.3 ± 8.1 a	32.9 ± 9.6 a
	Bacteroidia	0.2 ± 0.1 b	0.4 ± 0.3 ab	0.5 ± 0.2 ab	1.8 ± 0.7 a
	Actinobacteriota	50 ± 4.7 a	25.6 ± 4.5 b	30.6 ± 5.3 ab	25.8 ± 5.4 b
	Bacilli	0.9 ± 0.1 a	0.7 ± 0.1 a	0.5 ± 0.1 a	0.6 ± 0.1 a
90d	Alphaproteobacteria	74.6 ± 7.8 a	87.4 ± 1.9 a	88.6 ± 1.9 a	82.2 ± 3.6 a
	Acidobacteriae	2.1 ± 0.9 a	0.8 ± 0.2 ab	0.9 ± 0.2 ab	0.5 ± 0.1 b
	Verrucomicrobiae	1.1 ± 0.4 a	0.4 ± 0.2 a	0.7 ± 0.2 a	0.5 ± 0.2 a
	Gemmatimonadetes	0.1 ± 0 a	0.2 ± 0.1 a	0.1 ± 0 a	0.2 ± 0.1 a
	Gammaproteobacteria	2.7 ± 0.6 a	5.4 ± 1.5 a	3.8 ± 0.5 a	7.3 ± 2.7 a
	Bacteroidia	0.1 ± 0 a	0.4 ± 0.2 a	0.5 ± 0.2 a	0.4 ± 0.2 a
	Actinobacteriota	17 ± 9.1 a	3.5 ± 0.7 a	4 ± 1.3 a	6.8 ± 1.8 a
	Bacilli	0.5 ± 0.1 a	0.4 ± 0.1 a	0.3 ± 0 a	0.5 ± 0.1 a

Note: Data are presented as mean ± standard error (SE). CK, 1/3, 1/2, and Full denote no straw return, 1/3 straw return, 1/2 straw return, and full straw return, respectively. Different lowercase letters (a, ab, b) within the same row and the same period (30d or 90d) indicate significant differences among treatments ($P < 0.05$, Kruskal-Wallis test followed by Dunn's post-hoc test with Benjamini-Hochberg correction). Data are based on four replicates (n = 4).

Table S3. Relative abundance of dominant fungal classes under different straw return rates at 30 and 90 days.

Time	Class	CK	1/3	1/2	Full
30d	Sordariomycetes	45.6 ± 4 a	51.6 ± 5 a	45.6 ± 5.4 a	50.6 ± 4.6 a
	Eurotiomycetes	14 ± 3.7 a	16.1 ± 6.3 ab	2.3 ± 0.7 c	3.6 ± 1.6 bc
	Dothideomycetes	0 ± 0 b	0.4 ± 0.2 ab	2.9 ± 2.4 a	0.1 ± 0 ab
	Pezizomycotina_cls_Incertae_sedis	0.1 ± 0 a	0 ± 0 a	0.1 ± 0.1 a	5.2 ± 3.1 a
	Unclassified_p_Ascomycota	6.4 ± 1.8 b	14.4 ± 4 b	34.3 ± 1.7 a	33.6 ± 8.2 a
	Agaricomycetes	24.1 ± 6.5 a	13.5 ± 5.2 a	13.7 ± 5.5 a	6.7 ± 4.9 a
	Tremellomycetes	0.1 ± 0.1 a	0.3 ± 0.2 a	0.2 ± 0.2 a	0 ± 0 a
	Leotiomycetes	9.6 ± 6.2 a	3.6 ± 2.9 ab	0.7 ± 0.4 ab	0 ± 0 b
90d	Sordariomycetes	51.7 ± 6.5 a	42.7 ± 12.4 a	44.5 ± 4.7 a	58 ± 12.4 a
	Eurotiomycetes	23.7 ± 9.2 a	7.2 ± 4.1 a	20.8 ± 6.7 a	9.4 ± 3.4 a
	Dothideomycetes	0 ± 0 a	0.1 ± 0.1 a	0 ± 0 a	0.1 ± 0 a
	Pezizomycotina_cls_Incertae_sedis	1.6 ± 0.7 a	0.1 ± 0.1 a	0.5 ± 0.5 a	2.2 ± 1.7 a
	Unclassified_p_Ascomycota	13.7 ± 5.8 a	22.6 ± 11.7 a	9.5 ± 2.1 a	10.2 ± 4.6 a
	Agaricomycetes	8.9 ± 2.4 b	26 ± 5.9 a	22.7 ± 3.6 a	12.2 ± 2.8 ab
	Tremellomycetes	0.1 ± 0 ab	0 ± 0 b	1.3 ± 0.6 a	0.2 ± 0.1 ab
	Leotiomycetes	0.3 ± 0.2 a	0.3 ± 0.1 a	0.6 ± 0.1 a	7.6 ± 6.3 a

Note: Data are presented as mean ± standard error (SE). CK, 1/3, 1/2, and Full denote no straw return, 1/3 straw return, 1/2 straw return, and full straw return, respectively. Different lowercase letters within the same row and the same period (30d or 90d) indicate significant differences among treatments ($P < 0.05$, Kruskal-Wallis test followed by Dunn's post-hoc test with Benjamini-Hochberg correction). Data are based on four replicates (n = 4).

Table S4. Results of two-way PERMANOVA testing the effects of straw return rate and sampling period on bacterial and fungal beta diversity.

Organism	Source	Df	R^2	F	*P*
Bacteria	Treatment	3	0.103	2.24	0.003
	Period	1	0.264	17.21	0.001
	Treatment:Period	3	0.141	3.05	0.001
	Residual	32	0.492		
Fungi	Treatment	3	0.088	1.8	0.019
	Period	1	0.31	18.89	0.001
	Treatment:Period	3	0.077	1.57	0.049
	Residual	32	0.525		

Note: Treatment indicates straw return rate (CK, 1/3, 1/2, and Full); Period indicates sampling period (30d and 90d); Treatment:Period indicates their interaction. Df represents the degrees of freedom; R^2 represents the proportion of variance explained by each factor; F represents the F-statistic. *P*-values were calculated based on 999 permutations using the adonis function based on Bray-Curtis distance matrices.
